# Prevalence, Pattern and Predictors of Child Sexual Abuse Among Senior Secondary School Students in Enugu Metropolis

**DOI:** 10.21315/mjms2021.28.4.13

**Published:** 2021-08-26

**Authors:** Onyinye Hope Chime, Chinonyelu Jennie Orji, Tonna Jideofor Aneke, Ijeoma Ngozi Nwoke

**Affiliations:** 1 Department of Community Medicine, Enugu State University Teaching Hospital, Enugu State, Nigeria; 2 Department of Community Medicine, Enugu State University College of Medicine, Enugu State, Nigeria

**Keywords:** child, child abuse, sexual, prevalence, students, schools

## Abstract

**Objective:**

Child sexual abuse (CSA) is a growing public health concern with health, academic and psychosocial implications. The aim of this study was to determine the prevalence, pattern and predictors of CSA among secondary school students.

**Methods:**

This was a cross-sectional study carried out among adolescents in four secondary schools in Enugu Metropolis, Nigeria. A pretested self-administered questionnaire was used to collect information from 325 adolescents and data was analysed with a significance level set at *P* ≤ 0.05.

**Results:**

The prevalence of CSA in this study was 116 (35.7%). While the majority 20 (40%) of the victims were forced to watch pornography, most of the perpetrators were neighbours 34 (29.3%). A higher proportion of the victims were abused once, 79 (68.1%); when they were between 12 and 18 years old, 62 (53.4%); and at home, 39 (33.6%). Grouped ages, whom the child lived with, father’s and mother’s education, and father’s occupation were statistically significant on bivariate analysis. Predictors of CSA were students in senior secondary school Class 2 (SSS2) and those whose fathers were employed.

**Conclusion:**

Our study revealed a high prevalence of CSA. Comprehensive sexuality education and legislative policies should be implemented to educate adolescents and deter perpetrators.

## Introduction

The World Health Organization (WHO) defines child sexual abuse (CSA) as ‘the involvement of a CSA that he or she does not fully comprehend, is unable to give informed consent to, or for which the child is not developmentally prepared and cannot give consent or that violates the laws or social taboos of society. CSA is evidenced by the activity between a child and an adult or another child and who by age or development is in a relationship of responsibility, trust or power, the activity being intended to gratify or satisfy the needs of the other person. This may include but is not limited to the inducement or coercion of a child to engage in any unlawful sexual activity; the exploitative use of a child in prostitution or other unlawful sexual practices; the exploitative use of children in pornographic performances and materials’ (1). This includes sexual behaviour such as touching of the breasts, buttocks and genitals, whether the victim is dressed or undressed, fellatio, cunnilingus and penetration of the vagina or anus with sexual organs or with objects as well as watching of pornographic photography (2, 3).

According to the WHO, adolescents are defined as young people between the ages of 10 and 19 years old (4). At this age, they have limited knowledge about the natural processes of puberty, sexual health, pregnancy or reproduction (5). It is known to be the time for rapid development of knowledge and skills, learning to manage emotions, relationships, acquire attributes and abilities that will be necessary for enjoying the adolescent years and assuming adult roles (6). Globally, adolescents are particularly vulnerable and contribute to a third of all new cases of human immunodeficiency virus (HIV) infections, high levels of violence, early marriage and low school attendance rate and enrollment than primary school children (7–9). During this period, they suddenly become aware of the tremendous changes that are taking place in their sex organs and begin to seek various avenues to get answers to their sexual interest (7–10).

Worldwide, people are gradually realising that the closed nature of school environments and their absence of accountability systems can predispose adolescents to sexual abuse especially in Africa (10, 11). A study done in Nigeria showed that majority of the perpetrators were friends, neighbours and family members (12). Most perpetrators of sexual abuse are usually males and are known to their victims (13). A study done in the United States of America documented that for every 2 min, an adolescent is sexually assaulted and on the average over 237,868 victims are sexually assaulted each year (14).

CSA is becoming a social and public health concern especially because of its short- and long-term effects (9, 15). These include transmission of HIV and other sexually transmitted infections (STIs), somatic and visceral injuries, unwanted pregnancy, obstructed labour, vesico-vaginal and recto-vaginal fistula (16). They are also associated with high-risk behaviour such as multiple sexual partners, prostitution, delinquency in later life, substance abuse and psychological problems like feeling of vulnerability, fear, shame, guilt, poor self-esteem and depression (17–19).

The prevalence of CSA globally is 18% for girls and 7.6% for boys (20). According to WHO, 1 in every 13 men and 1 in every 5 females suffered various forms of sexual violence during childhood (21). The worldwide prevalence of CSA ranges from 5% to 36% (20, 22). The highest prevalence of CSA is in Africa (34.4%) followed by Asia and Oceania (23.9%), then Europe (9.2%) (20). The true burden of CSA in Nigeria is unknown and is estimated to vary between 5% and 38% across different parts of the country (23–25). Due to cultural diversities, weak child protection, legal and health-care systems, the exact prevalence rates of CSA in Nigeria may be difficult to evaluate as the crime is usually covered up and never reported (9, 15). Survivors of CSA usually display a more self-destructive attitude and experience more suicidal ideation than those who have not been abused (15). Irrespective of the adoption of the Child’s Right Act by Nigeria in 2003, which protects children against sexual abuse and exploitation, it remains a crime that does not only occur in Nigeria but also all around the world in different settings across various socioeconomic backgrounds and is punishable under the law (26–27).

The aim of this study was to determine the prevalence, pattern and predictors of CSA among secondary school children in Enugu Metropolis. This will throw light on CSA in secondary schools in Enugu, Nigeria so as to help affected parents and children cope with the management and prevention of this social problem (16). This study will also help government to inform policy formulations and design interventions to reduce the burden of CSA in Enugu Metropolis (16).

## Methods

### Study Area

Enugu Metropolis is the capital of Enugu State, Nigeria (28). It comprises Enugu South, Enugu North and Enugu East Local Government Area (29). It has a population of 722,664 as at the 2006 census (28). It is mainly populated by immigrants especially rural-urban migrants and this urban town lies about 221 metre to 317 metre above mean sea level. (28) There are about 28 public secondary schools spread across the city (29).

### Study Design and Population

This was a cross-sectional descriptive study. About 325 adolescents were included in the study which was carried out between April and June 2019. Senior secondary school students (Grades 1–3) attending public secondary schools registered with the Enugu State Ministry of Education were included in the study. This particular group of students were chosen because they are considered to be mature enough to talk about their sexual abuse experiences. However, the senior secondary school system in Nigeria comprises mostly of middle and late adolescents and they make up majority of our study population (15). The schools are the New Haven Secondary School, the Federal Government College, the Model High Secondary School Amaechi Awkunanaw and the Army Day Secondary School in Enugu State, Nigeria.

### Sample Size Determination

The minimum sample size was calculated using Fischer’s formula (30). The prevalence of CSA for this study was calculated from the prevalence obtained from a previous population-based study conducted in Enugu State University Teaching Hospital Parklane, Enugu (31). Using the Fischer’s formula of *N* = *Z*^2^ × *PQ*/*d*^2^ where *N* = sample size, *Z* = confidence interval at 95% level of significance as 1.96, *P* = reference prevalent rate (this was 8.8%), *d* = precision rate (0.05), we obtained a sample size of 123 and when a 10% non-response rate was added, the minimum sample size came up to 136. However, we chose to increase this sample size significantly to make our study robust.

### Sampling Procedure

A multi-stage sampling technique was used for this study. The list of registered secondary schools in the Enugu Metropolis was obtained from the Enugu State Ministry of Education. There are 13 public schools in the metropolis, five in Enugu South Local Government Area (LGA) and four each in Enugu North and Enugu South LGAs. The four schools recruited into the study were proportionally selected from the three LGAs in the metropolis [Enugu South LGA (Model High Secondary School Amaechi and Army Day Secondary School), Enugu East LGA (New Haven Secondary School) and Enugu North LGA (Federal Government College)]. The sampling frame was obtained from the register of senior secondary school students in the chosen schools. The next step involved a proportional allocation of the population of these students from each class in each of the selected schools. A systematic sampling method was then used in the selection of the students. Students were recruited consecutively until the sample size was obtained.

## Data Collection and Analysis

Information was collected using a pre-tested self-administered questionnaire which was designed from previous studies. These questions were validated by professionals working with sexually abused children like clinicians, police officers and social workers by including all the questions that were considered relevant and appropriate for this subject. Confusing questions were discarded upon advice by the expert reviewers. It was written in English language as this was the medium of instruction in all institutions of learning in Nigeria. The questions were grouped under different sections to elicit information on sociodemographics, prevalence and pattern of CSA. This was distributed to the participants and collection was made on the same day by the researcher. Information was collected from April to June 2019.

Data was analysed using Statistical Package for the Social Sciences version 22 software. Data analysis was presented in tables. Bivariate analysis was used in testing for the associations between CSA and sociodemographic characteristics. Furthermore, multivariate analysis was used to determine the predictors of CSA with the level of significance set at *P* ≤ 0.2.

## Ethical Clearance

Ethical approval was obtained from the Health Research and Ethics Committee of Enugu State University Teaching Hospital. A formal permission was requested and granted through the Commissioner of Education and Principals of each school. Signed consent and assent were obtained from parents/guardians of the respondents and the respondents themselves respectively. To maintain confidentiality of the respondents, serial numbers were used for identification instead of names; the questionnaires were kept safe properly and made easily obtainable to the researchers only. The researchers themselves described the purpose of the study to the respondents and were allowed to ask questions concerning the research before embarking on completion of the questionnaire (32). The researchers also explained to them that the study was risk free, however respondents who felt uncomfortable during the study were advised that they could withdraw at any point in time and directed to the school counselor for counseling.

## Results

Table 1 shows that out of 325 respondents, majority 230 (70.8%) were aged between 15 and 17 years old, 178 (54.8%) were females, most were from the Igbo tribe 306 (94.2%) and from senior secondary school Class 2 (SSS2) 187 (57.5%). More than half of the respondents were living with their parents 219 (67.3%). Secondary school education was the highest academic level achieved by of the fathers 263 (81.0%) and mothers 264 (82.2%).

**Figure 1:**
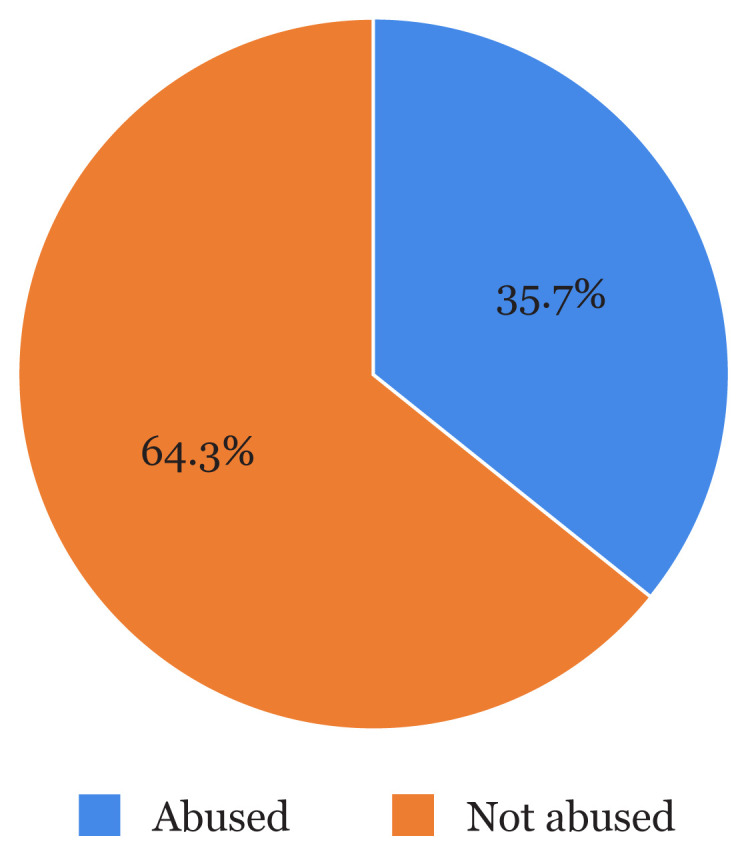
General prevalence of CSA among respondents

Figure 1 highlights the general prevalence of CSA among the respondents as 116 (35.7%).

Table 2 illustrates that out of a total of 116 respondents, 50 was for CSA without physical contact with majority of the group belonging to been forced to watch pornography 20 (40%). For those that were sexually abused with physical contact but without penetration, out of 45 respondents, 10 (22.2%) of them experienced been touched against will with sexual intentions and forced vaginal sexual intercourse against will but the abuser did not succeed. Out of a total of 21 respondents that experienced CSA with penetration, 8 (38.1%) of the respondents experienced forced vaginal intercourse.

Table 3 summarises the pattern of CSA among the respondents. Majority of the perpetrators were neighbours (34 [29.3%]), family member/boyfriend/girlfriend (20 [17.2%]) each and strangers (17 [14.7%]). Most of the respondents (62 [53.4%]) were abused between ages of 12 and 18 years old. Home (33.6%) was noted as the commonest place of abuse. While a higher proportion of the respondents experienced sexual abuse only once (68.1%) and still felt bad about the incident, (75 [64.5%]), a small proportion were still experiencing the abuse, (15 [12.9%]). Only 1.5% of the respondents reported sexual abuse to the authorities.

**Figure 2:**
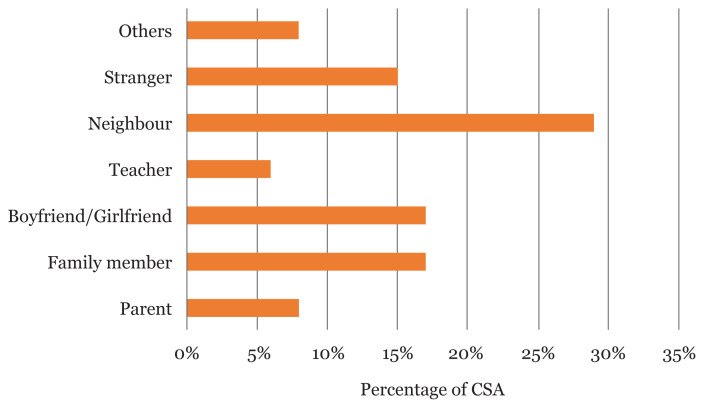
The identity of the CSA

Figure 2 reveals the identity of the abuser. Neighbours were found to be the commonest sexual abuser of children (34 [29.3%]).

**Figure 3:**
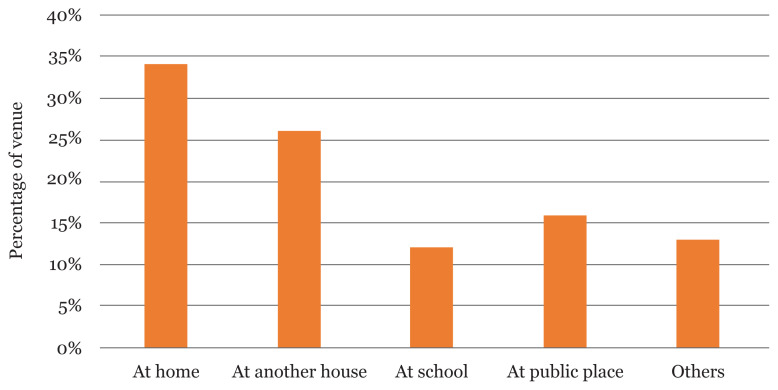
Common venues of CSA

Figure 3 shows that 39 (33.6%) CSA was most likely to occur at home.

Table 4 highlights the crude odds ratio estimate. Here, grouped ages, class of students, father’s occupation, mother’s and father’s education were noted to be individual factors that were associated with CSA. These variables were statistically significant at *P*-value of 0.05.

Table 5 highlights the results from logistic regression analysis. It reveals that the father’s education and class level of the students were significant predictors of CSA. Adolescents in SSS2 were 2.5 times more likely to experience sexual abuse than those in other classes. Fathers who had obtained at least a secondary education were 5 times more likely to have sexually abused children than other children. This result was found to be statistically significant.

## Discussion

The prevalence of CSA in our study was high (35.7%). This was similar with reports from other studies done in South-East Nigeria, South-South Nigeria, North-East Nigeria and Switzerland where the prevalence rates were 40%, 36.7%, 36% and 35.1%, respectively (14, 33–35). In contrast, a much lower prevalence rate at 0.06%, 0.4% and 1.6% were found in studies done in North-West Nigeria, Senegal and South Africa, respectively (15, 36, 37). This low prevalence rates can be attributed to different methodologies like the study area and the age of the study population (33, 38). Also in contrast, a higher prevalence rate of 69.9% and 77% were found in studies done in different parts of Nigeria though this was done among adolescents who were street hawkers and girls in paid employment (25, 39). This various discrepancies in the prevalence rates may be attributed to social and cultural variations found across different geographical zones in the world which can exhibit positive differences in incidence or are affected by how the disclosure and reporting of cases are understood in various cultures (33, 39). The Child Right Act of Nigeria enacted in 2003 provides the protection of children from CSA and carries a sentence of life imprisonment if the sexual offender is convicted. However, in 2015 the Violence Against Persons (Prohibition) Act was established and consisted of various levels of punishment ranging from life imprisonment to a minimum sentence of 12 years imprisonment depending on the type of sexual violence (40). However, this law has not been fully implemented. Educational programmes should be provided to parents and the community at large by the government. Child right advocates and practitioners must rise up and expound on the dangers of non-reporting of CSA as this may send a wrong signal to perpetrators to continue to abuse children (40).

In our study, females (60.3%) were more sexually abused than males. This was similar to findings from studies done among female students in Zaria, Nigeria and Ghana where over 80% of the victims were females (41,42). Studies carried out among university and high school students in Tanzania and Turkey were in concordance with our study that most of the victims were females at 31% and 13.4%, respectively (43, 44). This could be due to the fact that females are more vulnerable to sexual abuse because they are usually being used as domestic servants and victims of child labour (42). Another reason may be because females exhibit early sexual maturation compared to males and these physical features make them appear attractive, more vulnerable to unwanted advances, seduction by older and more experienced males (45). Males, on the other hand, are believed to contribute to this as it is known in the African setting due to their male ego, they hardly mention that they were sexually abused unlike females (46). In contrast, studies done in Saudi Arabia among secondary school students showed that sexual abuse occurred more in boys than girls (39, 47). However, study done in Lebanon among secondary school students showed no gender difference (48). This low prevalence of CSA among girls in Arab countries when compared to boys was attributed to their religion, cultural norms and values where girls are not allowed to go outside unattended making it difficult for them to be exposed to extra-familial perpetration though this does not affect the familial perpetration which is responsible for the majority of the CSA recorded in the Arab countries (49).

The commonest form of abuse was been forced to watch pornography (40%). Likewise a study done among secondary school students in Rivers State, Nigeria supports our findings that the most common form of abuse (32.4%) was watching pornographic materials like pictures, drawings, video tapes or magazines (33). In contrast, studies done in Ethiopia, Malawi, Nepal and Switzerland among adolescents showed that the most prevalent form of sexual abuse were verbal sexual harassment (32.2%), child been fondled (87.7%), the use of vulgar words (66.7%) and sexual harassment via internet (37.9%), respectively (35, 50–52). Pornography was found to be common in our study maybe because we are in the era of advanced technological know-how which gives gullible adolescents easy access to the social media through the internet of things bringing the perpetrators of this crime close enough to deceive or lure their victims. Pornography is known to cause sexual arousal for perpetrators but there are no reports supporting if it would make them act on this arousal but it seems to be part of the constellation about what can cause them to abuse adolescents (33).

Majority (29.3%) of our victims reported neighbours as their main perpetrators. This is similar to a research carried out among adolescents in Ethiopia (16%), Tanzania (> 30%), Egypt (44.8%) and Singapore which showed that most of their perpetrators were neighbours (53, 56). In contrast a study done in South-East Nigeria among adolescents, they identified family members and relatives (63.8%) as the most common perpetrators (14). Likewise, a study done in Port Harcourt shows that majority of the perpetrators were caregivers/family relatives (33.8%) (33). Strangers however were pointed out as the main perpetrators in reports from some Asian countries notably Bangladesh and Japan (57). Furthermore, a study conducted in South Korea reported peers as majority (20.7%) of the perpetrators in all research cases (58). This discrepancy may be due to environmental or cultural factors or both influencing the perpetration of CSA (15). However, the familiarity between the perpetrator and its victim makes suspicion of sexual abuse by parents or passer-by difficult to detect (46). This is common among children who were abused by family members and tend to withhold information regarding to their family members (45). Most of the sexually abused adolescents do not tell anyone about their sexual abuse even when asked by parents or any other higher authority (45). When a family member is a perpetrator, the victims are afraid to disclose the news because they are usually threatened or they believe that it will hurt their parents or that they may not be believed. However, they are more willing to talk about perpetrators outside their family and seek justice (45).

The home was the commonest place of abuse in our study (33.6%). This is consistent with a study done among secondary school children in India showing that majority (53%) of adolescents were sexually abused at home (59). In contrast, a study done in Vietnam among school age children considered schools as the main place of abuse (60). Also, studies done in Egypt (48.9%), Nepal (81%) and in the rural area of Northern Cape, South Africa (81.6%) revealed that majority of their victims were sexually abused in a public place and this finding was attributed to the use of drugs especially in Northern Cape (52, 61, 62). A similar study conducted in a South-West Nigeria showed that the place of abuse had an even spread among homes, schools, neighbours’ house and others (15). A study done on CSA in sub-Saharan Africa corroborates the fact that children can be sexually abused and exploited in a variety of settings (63). Survey done in the United Kingdom revealed that the home which should be a safe place appears to be the most dangerous place for children (64).

Majority of the adolescents were sexually abused once. This was similar to a study done in South-West Nigeria where 67.8% of all cases of CSA occurred only once (15). Another study done in Malawi among female students showed that 76.6% were not regularly abused while 21.0% indicated they were regularly abused (51). A similar study done in Nepal supports our finding as 76.2% of the victims experienced sexual abuse once while 19.0% experienced it frequently (52). Reason for this is not clear but believed to be because the perpetrator may act on impulsively when an opportunity presents itself and decides afterwards not to repeat that act as they usually prey on easy targets and other vulnerable victims to minimise chances of exposure (15). Under-reporting of CSA by the victims can be a contributory factor especially if they were threatened by the perpetrators adding to an overall low frequency of reporting of the occurrence.

Bivariate analysis revealed a significant association between age, class, parental education and father’s occupation with the prevalence of CSA. It is important to recognise that in bivariate analysis, *P*-values and crude odds ratio are obtained which have not taken into cognizance, confounding variables. We can only generate predictors from only multivariable analysis (logistic regression) that takes care of variables that may be confounding the true association between an exposure and an outcome.

There was a significant association between age and prevalence of CSA on bivariate analysis. Adolescence is a rapid phase in human development with its biological maturity preceding its psychosocial maturity (65). Since they are still undergoing physical, neurodevelopmental and psychosocial changes, their inquisitiveness and eagerness to experiment new things make them easy target that can be lured by their perpetrators if not duly monitored by parents and guardians (65). It is pertinent that non-governmental organisations, schools, churches or religious leaders should be involved in providing sex education programmes for children to help protect them against sexual predators.

On multivariate logistic regression, class level and father’s education were found to be the only significant predictors of CSA since students in SSS2 were 2.5 times more likely to be sexually abused than students in other classes. However, we must state that this may not be a true association since the confidence interval for SSS2 crosses one. Students in high senior secondary classes are particularly vulnerable as a result of their ages and the stage of adolescence that they are undergoing and tend to be more adventurous especially in the absence of parental monitoring and supervision. This is made worse by the societal norms, pressures and weak policies that propagate the weak nature and sexual inferiority of the girlchild (10). Students in SSS2 usually have more time than those in SSS1 and SSS3. While students in SSS1 are trying to settle down after undergoing external junior secondary school examinations to get into SSS1, those in SSS3 on the other hand are busy preparing for their senior school certificate examinations as well as their university entrance examinations. There was no other study found to have any similar finding to ours in terms of class level and its association with CSA. More research maybe needed in this area. This study also revealed that a high proportion of the respondents whose parents achieved secondary school education and above were more likely to be sexually abused than others. This finding is in contrast to a study done among female university students in Eastern Ethiopia where achieving secondary school education by the father was associated with low sexual abuse (66). Likewise, respondents whose mothers attained secondary school education and above were more abused than those with primary school education and below. Generally, parents with higher educational qualifications tend to have better employment opportunities as they work outside the family setting, exposing their children to sexual abuse through lack of parental protection and supervision (45). Our study revealed that adolescents whose fathers were self-employed or salaried were more up to five times more likely to be sexually abused. This may be attributed to the fact that such fathers had better employment opportunities and so were away from home (45).

## Conclusion

This study showed a low prevalence of CSA with majority of the perpetrators were neighbours and the sexual abuse occurred mostly at home. The only predictor of the prevalence of CSA was found to be the SSS2 class. Efforts should be made to make sure that the children recognise abusive situations and are able to disclose to authorities who their abusers are. A stable home is important as this helps parents monitor who their children are with at home, including other family members, neighbours and domestic helps since most cases of CSA in our study happened at home. The government should provide public enlightenment on the risk factors and prevention of CSA to help combat its occurrence. Guidance and counseling units, religious groups could play a vital role in putting adolescents through all the available and accessible sex education sources with the aim of improving their reproductive health and making them live a better life. The Education Ministry as well as non-governmental organisations should be involved to help provide adequate resources for implementing education programme for secondary schools in Nigeria.

## Limitation of Study

CSA can be perceived as a taboo especially in Africa. This could have resulted in social desirability bias as the respondents may be reluctant to be fully honest about their actual experiences and the willingness to give correct information thereby underestimating the magnitude of the problem.

## Figures and Tables

**Table 1:** Sociodemographic characteristics of the respondents

Variables	Descriptions	*N* (%)
Age (years)	11–13	28 (8.6)
	14–16	230 (70.8)
	17–19	67 (20.6)
Gender	Male	147 (45.2)
	Female	178 (54.8)
Class	SSS1	86 (26.5)
	SSS2	187 (57.5)
	SSS3	52 (16.0)
Tribe	Igbo	306 (94.2)
	Others	19 (5.8)
Who do you live with	Both parents	219 (67.3)
	One parent	46 (14.2)
	Relatives	40 (12.3)
	Non-relatives 20	(6.2)
Mother’s education level	Primary education and below	58 (17.8)
	Secondary education and above	264 (82.2)
Father’s education level	Primary education and below	62 (19.0)
	Secondary education and above	263 (81.0)
Mother’s occupation	Unemployed	13 (4.0)
	Student	13 (4.0)
	Self-employed	177 (54.5)
	Salary-employed	117 (36.0)
	Retired	5 (1.5)
Father’s occupation	Unemployed	15 (4.6)
	Student	16 (4.9)
	Self-employed	160 (49.2)
	Salary-employed	110 (33.9)
	Retired	24 (7.4)

**Table 2:** Prevalence of CSA among the respondents

Variables	*N* (%)
CSA without physical contact (*n* = 50)	
Forced to look at the genitalia	10 (20)
Forced to show naked body	9 (18)
Forced to watch pornography	20 (40)
Pictures of nude body taken against will	7 (14)
Shared pictures of self to others against will	4 (8)
CSA with physical contact but without penetration (*n* = 45)	
Touched against will with sexual intention	10 (22.2)
Forced to kiss someone	5 (11.1)
Forced penetration with finger, someone tried to but did not succeed	10 (22.2)
Forced vaginal intercourse against will, someone tried but did not succeed	8 (17.8)
Forced anal intercourse, someone tried to but did not succeed	5 (11.1)
Forced oral intercourse, someone tried to but did not succeed	5 (11.1)
Forced into prostitution	2 (4.5)
CSA with penetration (*n* = 21)	
Forced penetration with finger or object	7 (33.3)
Forced vaginal intercourse	8 (38.1)
Forced anal intercourse	3 (14.3)
Forced oral intercourse	3 (14.3)

**Table 3:** Pattern of CSA among the respondents

Variables	Descriptions	*N* (%)
Identity of abuser	Parent	9 (7.8)
	Family member	20 (17.2)
	Boyfriend/Girlfriend	20 (17.2)
	Teacher	7 (6.0)
	Neighbour	34 (29.3)
	Stranger	17 (14.7)
	Others	9 (7.8)
Age at first abuse	1–6 years old	234 (72.0)
	7–11 years old	42 (12.9)
	12–18years old	49 (15.1)
Place where event occurred	At home	39 (3.6)
	At another home	30 (25.9)
	At school	14 (12.1)
	At public place	18 (15.5)
	Others	15 (12.9)
Frequency of occurrence	Once	79 (68.1)
	2–5 times	27 (23.3)
	6–10 times	5 (4.3)
	More than 10 times	5 (4.3)
Still experiencing abuse	Yes	15 (12.9)
	No	101 (87.1)
Still feel bad about the incident	Yes	75 (64.7)
	No	41 (35.3)
Reported to the authorities	Yes	15 (1.5)
	No	101 (98.5)

**Table 4:** Bivariate analysis of CSA among the respondents

Variables	Yes (*n* = 116)	No (*n* = 209)	Chi-squared (*χ*^2^)	*P*-value
Gender				
Male	46 (39.7)	101 (48.3)	2.264	0.132
Female	70 (60.3)	108 (51.7)		
Age group (years old)				
11–13	5 (4.3))	23 (11.0)	11.109	0.004*
14–16	95 (81.9)	135 (64.6)		
17–19	16 (13.8)	51 (24.4)		
Class				
SSS1	8 (6.9)	78 (37.3)	61.203	< 0.001*
SSS2	100 (86.2)	87 (41.6)		
SSS3	8 (6.9)	44 (21.1)		
Tribe				
Igbo	111 (95.7)	195 (93.3)	0.773	0.379
Others	5 (4.3)	14 (6.7)		
Who they live with				
Non-relatives	4 (3.4)	16 (7.7)	2.286	0.131
Parents/Relatives	112 (96.6)	193 (92.3)		
Mother’s education				
Primary education and below	9 (7.8)	49 (23.4)	12.520	< 0.001*
Secondary and above	107 (92.2)	160 (76.6)		
Father’s education				
Primary education and below	7 (6.0)	55 (26.3)	19.876	< 0.001*
Secondary education and above	109 (94.0)	154 (73.7)		
Mother’s occupation				
Unemployed	4 (3.4)	14 (6.7)	1.506	0.220
Employed	112 (96.6)	195 (93.3)		
Father’s occupation				
Unemployed	9 (7.8)	33 (15.8)	4.275	0.039*
Employed	107 (92.2)	176 (84.2)		

Note:

* Signifies significant variables entered into logistic regression

**Table 5:** Predictors of CSA among the respondents

Variables	Wald	*P*-value	AOR (95% CI on multivariate analysis)
Sex (female)	1.378	0.240	1.401 (0.798–2.459)
Class			
SSS1	46.243	< 0.001	1
SSS2	2.297	0.130	2.482 (0.766–8.045)
SSS3	13.997	< 0.001	0.181 (0.074–0.444)
Graded age (years old)			
11–13	1.589	0.452	1
14–16	0.341	0.559	0.653 (0.156–2.736)
17–19	1.589	0.207	0.613 (0.286–1.312)
Mother’s education (secondary and above)	1.097	0.295	1.643 (0.649–4.159)
Father’s education (secondary and above)	11.728	0.001	5.264 (2.035–13.618)
Father’s occupation (employed)	2.790	0.095	2.126 (0.877–5.150)
Living with relatives	2.612	0.106	2.819 (0.802–9.902)

Notes: AOR = adjusted odds ratio; *P*-value of 0.2 was used as a cut-off point from which variables were moved from bivariate into multivariate analysis
